# Drug repositioning of disulfiram induces endometrioid epithelial ovarian cancer cell death via the both apoptosis and cuproptosis pathways

**DOI:** 10.32604/or.2023.028694

**Published:** 2023-05-24

**Authors:** YAPING GAN, TING LIU, WEIFENG FENG, LIANG WANG, LI LI, YINGXIA NING

**Affiliations:** 1 Department of Gynaecology and Obstetrics, The First Affiliated Hospital of Jinan University, Guangzhou, China; 2 Department of Gynaecology and Obstetrics, The First Affiliated Hospital of Guangzhou Medical University, Guangzhou, China; 3 Department of Anesthesiology, Ruijin Hospital, Shanghai Jiao Tong University School of Medicine, Shanghai, China; 4 Guangdong Guojian Pharmaceutical Consulting Co., Ltd., Guangzhou, China; 5 Department of Galactophore Surgery, The Second Affiliated Hospital of Nanchang University, Nanchang, China

**Keywords:** Ovarian cancer, Drug repositioning, Disulfiram, Apoptosis, Cuproptosis

## Abstract

Various therapeutic strategies have been developed to overcome ovarian cancer. However, the prognoses resulting from these strategies are still unclear. In the present work, we screened 54 small molecule compounds approved by the FDA to identify novel agents that could inhibit the viability of human epithelial ovarian cancer cells. Among these, we identified disulfiram (DSF), an old alcohol-abuse drug, as a potential inducer of cell death in ovarian cancer. Mechanistically, DSF treatment significantly reduced the expression of the anti-apoptosis marker B-cell lymphoma/leukemia-2 (Bcl-2) and increase the expression of the apoptotic molecules Bcl2 associated X (Bax) and cleaved caspase-3 to promote human epithelial ovarian cancer cell apoptosis. Furthermore, DSF is a newly identified effective copper ionophore, thus the combination of DSF and copper was used to reduce ovarian cancer viability than DSF single treatment. Combination treatment with DSF and copper also led to the reduced expression of ferredoxin 1 and loss of Fe-S cluster proteins (biomarkers of cuproptosis). *In vivo*, DSF and copper gluconate significantly decreased the tumor volume and increased the survival rate in a murine ovarian cancer xenograft model. Thus, the role of DSF revealed its potential for used as a viable therapeutic agent for the ovarian cancer.

## Introduction

Ovarian cancer is a highly lethal malignancy that affects the reproductive system of females globally [1,2]. Findings from surveillances and epidemiology and end results process, it is suggested that 1.2% of women developing ovarian cancer during their lifetimes [3]. Ovarian carcinoma also accounts for a large number of deaths worldwide, and the mortality resulting from ovarian cancer is increasing at an alarming rate [4]. Over 50% of patients with ovarian cancer remain asymptomatic, leading to disease detection at advanced stages [5]. An additionally, the 5-year survival rate of late-stage ovarian cancer patients (25%) is significantly lower than for those diagnosed at stage I (92%) [6]. Although ovarian cancer shows resistance and high recurrence rate to chemotherapy [7], the only available complete treatment is surgery. Drug repositioning is a cost-effective approach to make approved drugs available for clinical treatment rapidly [8]. Prior investigation into the repositioning of drugs, such as trimebutine maleate and deferasirox, has demonstrated their ability to treat cancer by targeting ovarian cancer stem cells or ovarian cancer cell apoptotic activity [9,10]. Disulfiram (DSF) is an old alcohol-abuse drug that was approved by the US Food and Drug Administration (FDA) in 1951 [11]. Except for its activity as an acetaldehyde dehydrogenase (ALDH), long term DSF therapy has shown no toxicity towards animals over the years [12,13]. Recent studies have revealed the use of DSF in various diseases, including anti-tumor activity and resistance to sepsis and obesity [14–16]. Most studies have found that DSF exerts its anticancer effects by suppressing the tumor cell cycle, and cell proliferation and inducing tumor cell apoptosis. The precise mechanisms underlying the anticancer ability of DSF are still not fully understood. Furthermore, an increasing number of studies have demonstrated that DSF exhibits significant antitumor activity in a variety of cancers, including breast cancer [16], glioblastoma [17], prostate cancer [18], pancreatic cancer [19], melanoma and acute myeloid leukemia [19]. However, the role of DSF in ovarian cancer is unknown.

When DSF consumed orally, it is metabolized into dithiocarbamate (DTC). The DTC moieties of DSF can chelate bivalent metals, especially copper ions (Cu^2+^), to form the DSF/Cu complex [16,20]. Copper plays an important role in the generation of ROS in tumor cells [21–23]. As products of mitochondrial oxidative phosphorylation, ROS play an essential role in metabolic and other biological events, by acting as intracellular signaling molecules or toxic byproducts of aerobic metabolism. Over accumulation of ROS promotes lipid, protein and DNA damage, which subsequently causes cell death [24,25]. Recently, DSF was identified as an effective copper ionophore, and treatment of lung cancer cells with DSF and copper was found to cause a new form of cell death called cuproptosis [23].

In the present study, using a screening system for drug repositioning, we identified that DSF significantly inhibits the viability of human epithelial ovarian cancer cells. We further uncover a novel mechanism whereby DSF/Cu complex dependent ovarian cancer cell cuproptosis. We also explore the therapeutic potential of targeting this pathway in the context of ovarian cancer.

### The screening system for repositioning of natural compounds as drugs revealed that disulfiram suppressed the viability of human epithelial ovarian cancer cells

**Figure 1:**
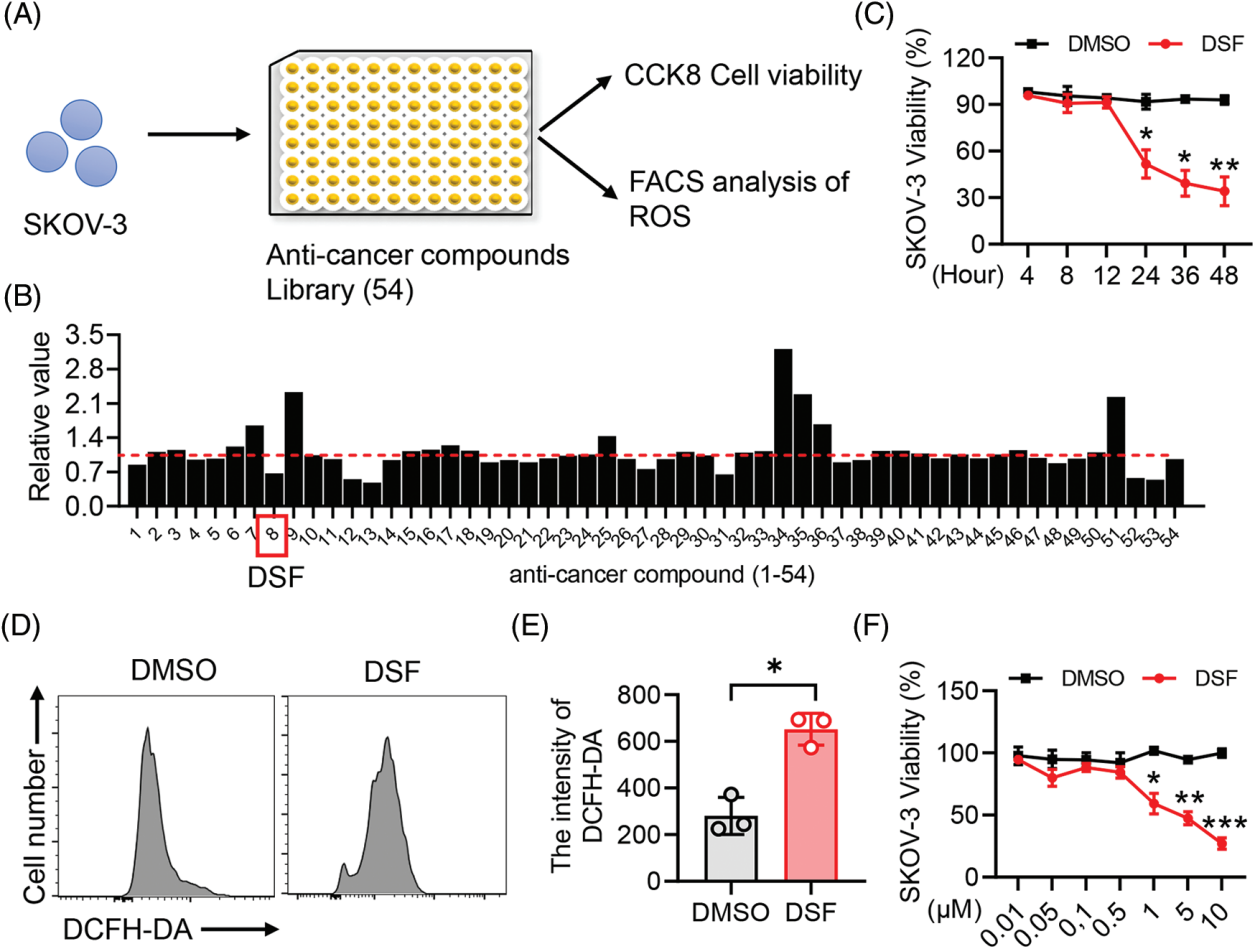
DSF promoted SKOV-3 cancer cell apoptosis. (A) CCK8 and Fluorescence activated cell sorting (FACS) screening assay in SKOV-3 cells. (B) Cell Counting Kit-8 (CCK8) analysis of cell viability of SKOV-3 cells treated with the screened anti-cancer compounds for 24 h. (C) FACS analysis of Reactive oxygen species (ROS) in SKOV-3 cells treated with Dimethylsulfoxide (DMSO) or disulfiram (DSF) (5 µM) for 24 h. (D) Quantitative analysis of 2,7-Dichlorodihydro fluorescein diacetate (DCFH-DA) intensity in (C). (E) CCK8 analysis of cell viability of SKOV-3 cells treated with DSF (5 µM) for indicated time durations. (F) CCK8 analysis of cell viability of SKOV-3 cells treated with DSF at indicated concentrations for 24 h. Data in all panels are representative of at least three ((B–D) n = 3, and (E, F) n = 5, mean ± SD) independent experiments. *p < 0.05; **p < 0.01; ***p < 0.001 (for comparing two groups, unpaired Student’s *t* test was used in (B) and (D) and two-way analysis of variance [ANOVA] was used in (E) and (F)).

Drug repositioning is a good strategy for lowering time and costs and improving success rates for drug development. Drug repurposing strategies can be divided into two types, including activity-based drug repositioning and computational drug repositioning. Herein, we used an activity-based drug repositioning strategy to screen 54 natural small molecular compounds and analyze their effects on the viability of human epithelial ovarian cancer SKOV-3 cells. To this end, Human epithelial SKOV-3 ovary cancer cells were plated in a 96-well plate. The cells were then treated with the indicated anti-cancer compounds for 48 h, and cell viability were detected using the CCK8 assay (Fig. 1A). The results showed that DSF could strongly inhibit the viability from human epithelial SKOV-3 cells compared to the other 53 candidates (Fig. 1B). To identify the cytotoxic activity of DSF against ovarian cancer cells, we examined eugenyl acetate (negative control), cisplatin (positive control), and DSF. DSF exhibited a significant cytotoxic effect against both SKOV-3 and A278 cells compared with that against HEK293 cells (Table 1). The anticancer activity of DSF involves several mechanisms dependent on elevated intracellular reactive oxygen species (ROS) levels [26]. Our results showed a significant increase in the DCFH-DA intensity of SKOV-3 cells after DSF treatment (Figs. 1C and 1D), indicating the role of DSF in ROS production in SKOV-3 cells. Furthermore, we detected the effects of different concentrations of DSF on the cell viability of SKOV-3 cells at different treatment durations. It was found that 5 μM DSF suppressed SKOV-3 cell viability at 24 h and significantly suppressed it at 48 h (Fig. 1E). Meanwhile, 1 and 10 μM DSF significantly suppressed SKOV-3 cell viability at 24 h (Fig. 1F). These findings indicated that DSF suppressed the viability of human epithelial SKOV-3 cells at a time and dose-dependent manner.

**Table 1:** Cytotoxic activities of DSF, eugenyl acetate and cisplatin in human epithelial ovarian cancer cells (SKOV3 and A2780) and normal kidney cells (HEK293)

		^a^IC50 (μM) (95% Cl)		
Compound	Name	SKOV3	A2780	HEK293
1	Disulfiram	21.13 (20.01–22.25)	19 (17.37–20.63)	6.56 (5.87–7.26)
2	Eugenyl acetate	>200	179.84 (162.177–197.51)	>200
3	Cisplatin	3.45 (2.90–4.13)	12 (9.81–14.55)	3.56 (2.46–4.91)

Note: ^a^Concentrations that show 50% cell viability inhibition. Cl: confidence interval.

### DSF significantly suppressed the tumor volume and increased the survival rate in an ovarian tumor xenograft mouse model

**Figure 2:**
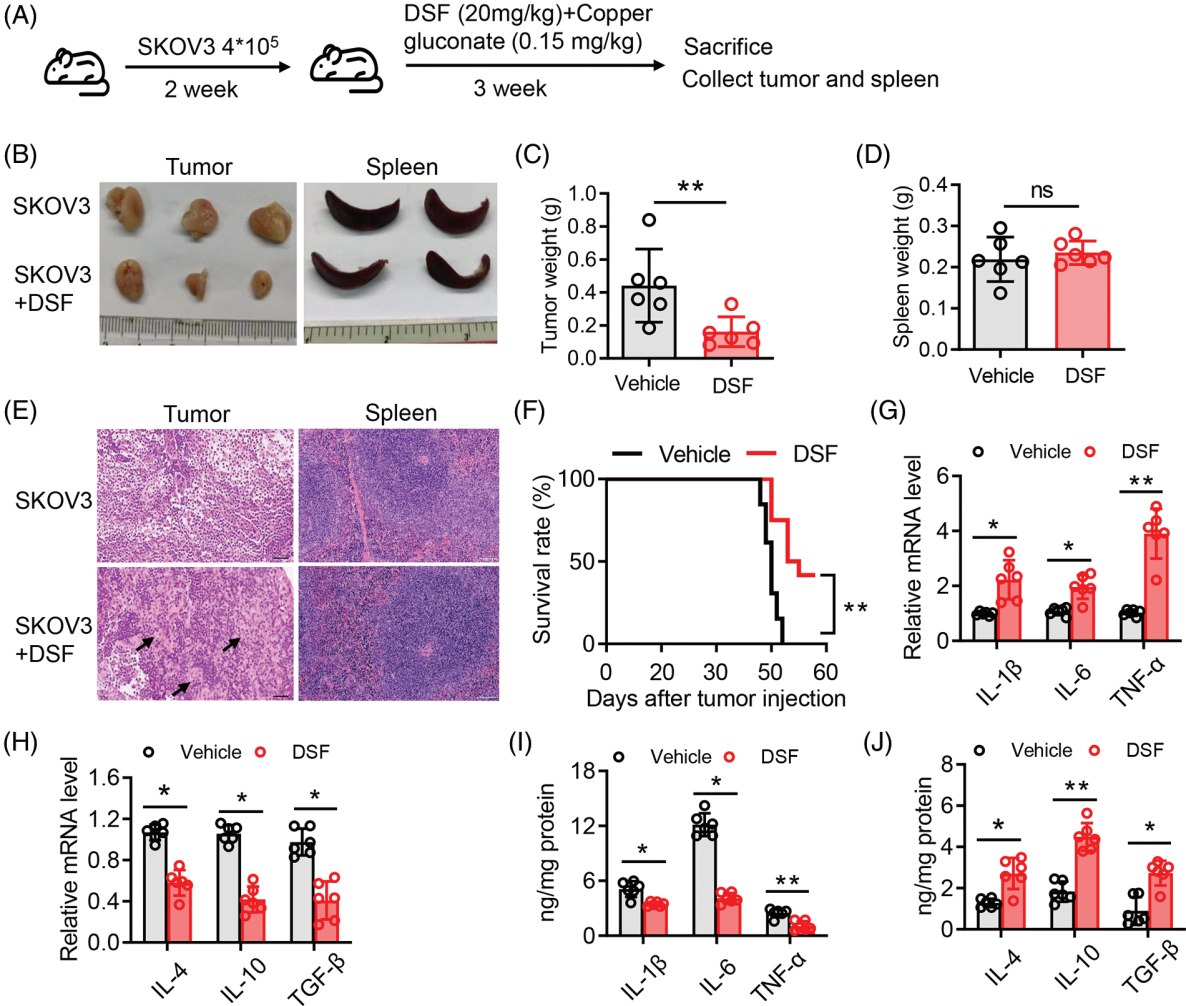
DSF suppressed tumor growth in an ovarian tumor xenograft mouse model. (A) Schematic depicting the treatment of the SKOV-3 cell-based tumor-bearing mouse model for assessing the anti-cancer effect of DSF. DSF (20 mg kg^−1^, suspended in 0.5% NaCMC), gluconate (0.15 mg kg^−1^), and vehicle (equal volume of 0.5% NaCMC)-treated mice after SKOV-3 ovarian cancer cell inoculation (n = 5 mice per group). (B) Photographs of xenograft tumors and spleens from NOD/SCID mice injected with SKOV-3 cells at day 30. (C and D) Quantitative analysis of the weights of tumors (C) and spleens (D) from mice treated with the vehicle or DSF. (E) H&E staining of tumor and spleen from mice treated with the vehicle or DSF. Scale bar, 100 μm. (F) Survival rate of tumor-bearing mice after treatment with vehicle or DSF. (G) The mRNA expression levels of IL-1β, IL-6 and TNF-α isolated from tumors in mouse administrated with vehicle or DSF (20 mg kg^−1^) at day 30 after ovary inoculation. (H) The mRNA expression levels of IL-4, IL-10 and TGF-βfrom tumors in mice treated with vehicle or DSF (20 mg kg^−1^) at day 30 after SKOV-3 cell inoculation. (I) ELISA data of IL-1β, IL-6 and TNF-α isolated from tumors in mouse administrated with Vehicle or DSF (20 mg kg^−1^) at day 30 after ovary inoculation. (J) ELISA data of IL-4, IL-10 and TGF-β isolated from tumors in mouse administrated with Vehicle or DSF (20 mg kg^−1^) at day 30 after ovary inoculation. Data in all panels are representative of at least three ((C–J) n = 5, mean ± SD) independent experiments. *p < 0.05; **p < 0.01 (unpaired *t* tests for measurements between the two groups in (C) and (D). Two-way ANOVA was used in (G–J) and log-rank (Mantel-Cox) test was used in (F)).

We next sought to explore the effect of DSF on ovarian tumors in a murine xenograft mouse model. To this end, SKOV-3 cells (1.25 × 10^6^) were injected intraperitoneally into NOD/SCID mice. The mice were then divided into two groups, and copper gluconate was administered to them each morning (8 am) and DSF each evening (7 pm) (Fig. 2A). Body weight measurements were taken once every 3 days. The tumor volume was found to be significantly suppressed at 15 days after SKOV-3 injection. The tumor weight was significantly decreased after DSF treatment (Figs. 2B and 2C), while the weight of the spleen showed no change (Figs. 2B and 2D). H&E showed a reduced cell nuclear stating in DSF group compared with vehicle, while the staining of spleen showed no change between the two groups (Fig. 2E). We also assessed the survival rate of the ovarian tumor xenograft mouse model. The survival rate of tumor-bearing mice was significantly reduced at 48 days, but it was still over 50% after 55 days (Fig. 2F). It is crucial to comprehend the impact of oxidative stress-dependent pro-inflammatory cytokines in the tumor microenvironment [27]. Thus, we next assessed pro-inflammatory cytokines in ovarian tumors. Ovarian tumors were isolated from the tumor-bearing mice, and mRNA was extracted from them using TRIzol. Results of qPCR performed using this mRNA showed that the levels of proinflammatory cytokines, including IL-1β, IL-6, and TNF-α were significantly increased in tumor-bearing mice after DSF treatment (Fig. 2G). Increased anti-inflammatory cytokine levels are also known to significantly contribute to tumor progression [28]. In the present study, compared to those of pro-inflammatory cytokines, the levels of anti-inflammatory cytokines including IL-4, IL-10 and tumor growth factor-β (TGF-β) were suppressed in tumor-bearing mice after DSF treatment (Fig. 2H). We further detected protein level of these cytokines. The level of IL-1β, IL-6 and TNF-α were increased in tumor mice after DSF treatment, while the level of IL-4, IL-10 and TGF-β were increased after DSF treatment (Figs. 2I and 2J). Collectively, these data indicated that DSF suppressed the progression of ovarian cancer.

### CuET significantly promoted cell apoptosis and inhibited cell cycle progression in human epithelial ovarian tumor cells

**Figure 3:**
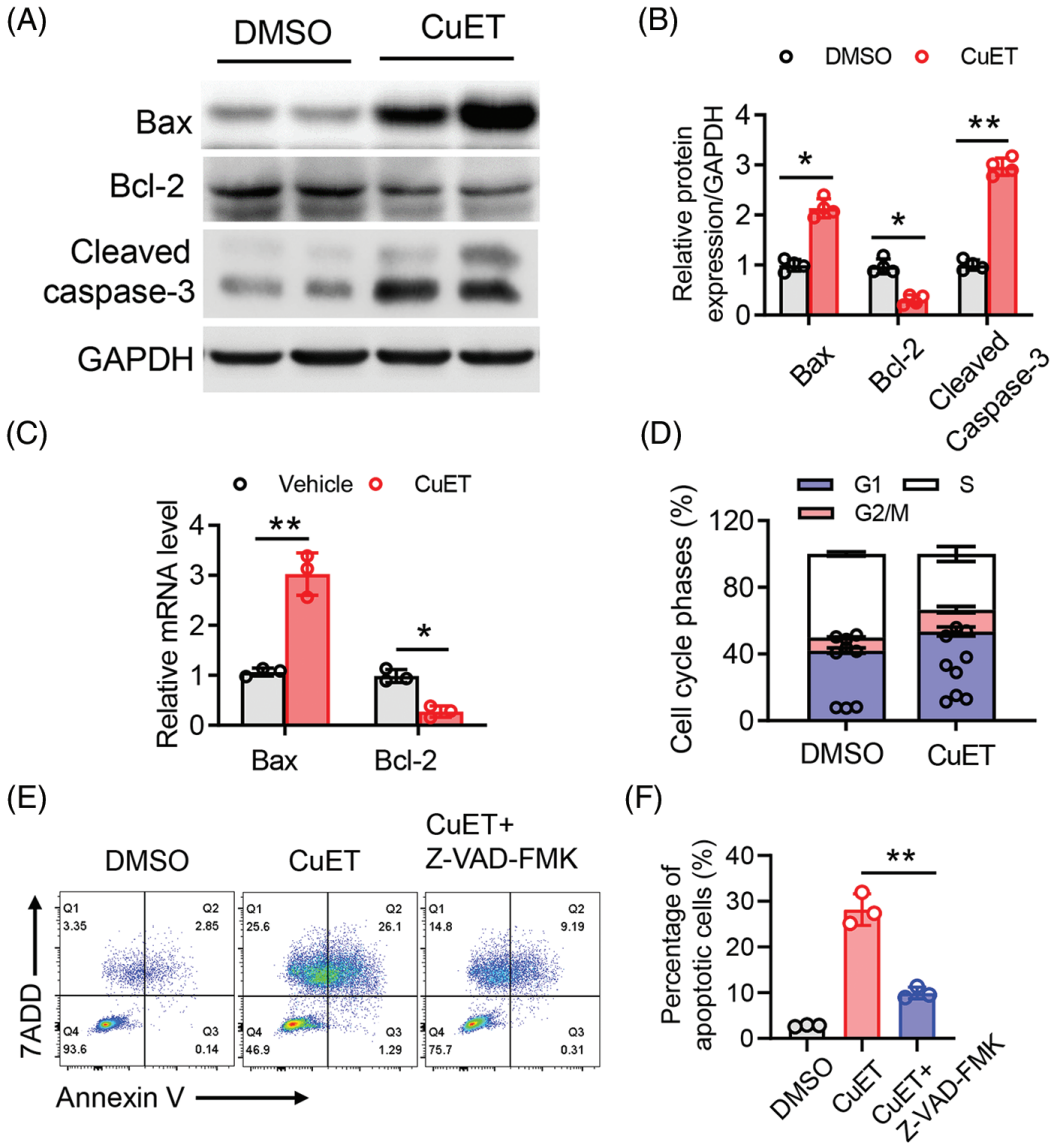
CuET promoted apoptosis in SKOV-3 cells. (A) and (B) Western blotting of Bax, Bcl-2, pro-caspase-3, cleaved caspase-3, as well as GAPDH in SKOV-3 cells treated with the DMSO or CuET (1 µM) for 24 h. (C) qPCR analysis of Bax and Bcl-2 in SKOV-3 cells treated with DMSO or CuET (1 µM) for 24 h. (D) FACS analysis of cell cycle phases of SKOV-3 cells treated with DMSO or CuET (1 µM) for 24 h. (E) FACS profiles of Annexin V-APC/7-AAD staining of SKOV-3 cells undergoing apoptosis by CuET or CuET with Z-VAD-FMK pretreatment. (F) Quantitative analysis of the percentage of apoptotic cells in (E). *p < 0.05; **p < 0.01 (two-way ANOVA in (B) and (C), one-way ANOVA in (F)).

DSF, dithiocarbamate (DDTC) and pyrrolidine dithiocarbamate (PDTC) are members of the dithiocarbamate family are Cu chelators. Once absorbed into blood, both DSF and the DSF/Cu complex are degraded to DDTC rapidly [29]. Therefore, we next added a ditocarbamate-copper complex DTC-copper and CuET into an *in vitro* culture system. Taken together, the results demonstrated that RES suppressed ovarian tumor cell metastasis by reshaping the tumor immune microenvironment. Relative levels of the apoptotic proteins, Bax, Bcl-2 and cleaved caspase-3 were then determined using western blotting. Compared to that in the control group, after CuET exposure at 5 μM for 24 h, the level of Bcl-2 was significantly decreased compared with that in the DMSO group, while the levels of Bax and cleaved caspase-3 were increased compared with those in the DMSO group (Figs. 3A and 3B). We also assessed the mRNA expression levels of Bax and Bcl-2 thrgouh qPCR and found that they were significantly increased after CuET treatment (Fig. 3C). To further conduct the effect of DSF on ovarian cancer cell death and growth. We used flow cytometry to detect tumor cells in the G1, G2 and M phase of cell cycle. The results showed that cells in the G2/M plus S phases were reduced after DSF treatment (Fig. 3D). Annexin V-APC/7AAD staining, a highly sensitive method for detecting apoptotic cells [30], was used to determine whether DSF or CuET could induce ovarian tumor cell apoptosis. As shown in Fig. 3, 27.39% of cells became apoptotic (early apoptosis plus late apoptosis) upon incubation with CuET, and this apoptotic effect of CuET was significantly suppressed by the pan caspase inhibitor Z-VAD-FMK (Figs. 3E and 3F). Collectively, the results indicated that DSF and CuET significantly promotes human epithelial ovarian tumor cell apoptosis.

### CuET significantly suppressed cuproptosis-related genes in ovary tumor cells

**Figure 4:**
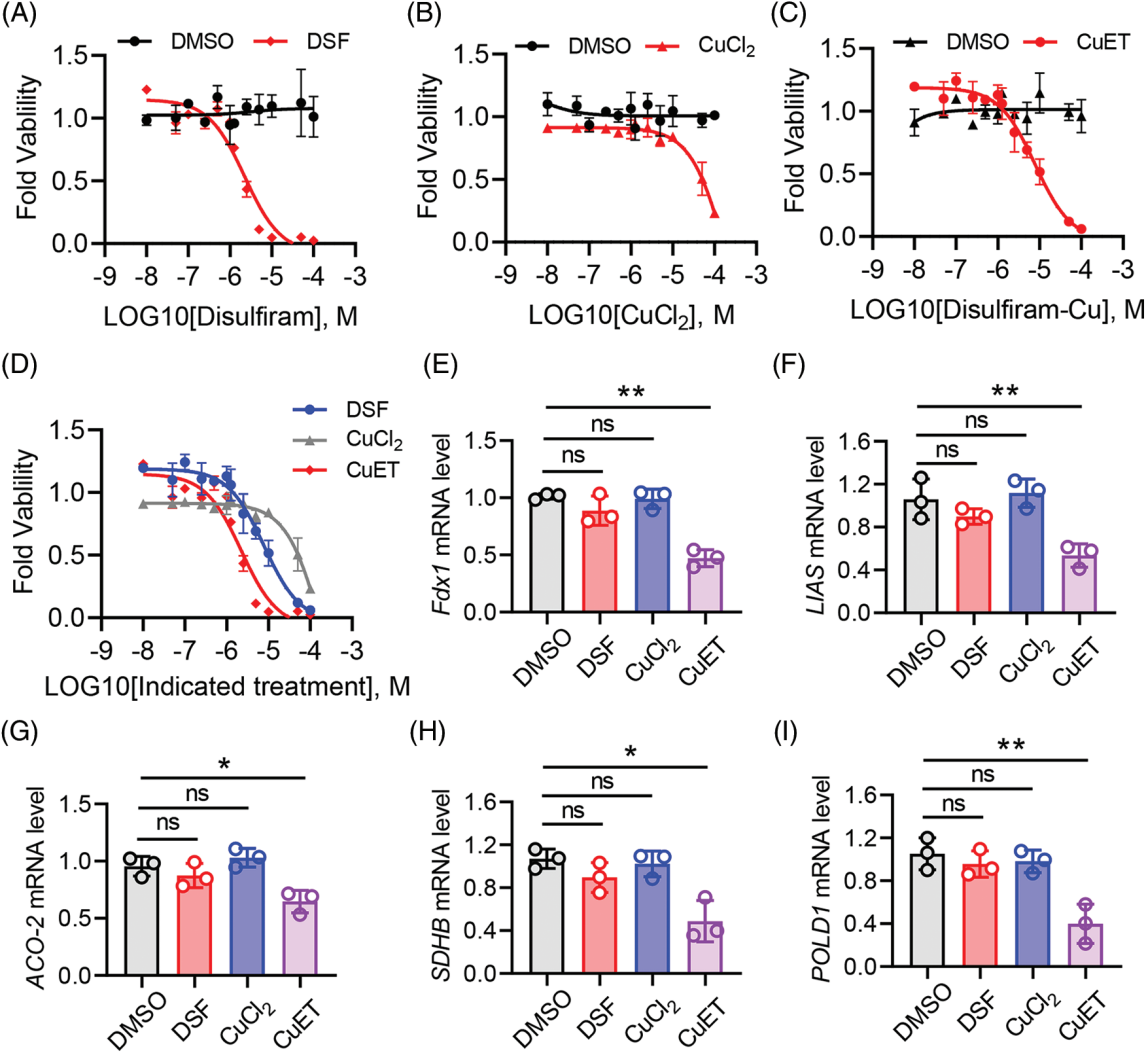
CuET induced SKOV-3 cell death through cuproptosis. (A–C) Quantitation of SKOV-3 cell death after 48 h of treatment with increasing doses of DSF (A), CuCl_2_ (B) and CuET (C). (D) Quantitation of SKOV-3 cell death after 48 h of combined treatment with increasing doses of DSF, CuCl_2_ and CuET. (E–I) mRNA expression levels of fdx1 (E), LIAS (F), ACO-2 (G), SDHB (H) and POLD1 (I) in SKOV-3 cells after 48 h of treatment with increasing doses of DSF, CuCl_2_ and CuET. Data in all panels are representative of at least three ((A–D) n = 4, and (E and F) n = 3, mean ± SD) independent experiments. *p < 0.05; **p < 0.01 (unpaired Student’s *t* test was for comparing two groups in (A–D) and one-way ANOVA was used for comparing more than two groups in (E–I)).

Recently, DSF was identified as an effective copper ionophore, and combination of DSF and copper treatment lead to a new form of cell death, cuproptosis [23]. Thus, we investigated the role of DSF, copper and CuET on cell death in human epithelial SKOV-3 cells. Treatment with DSF, copper and CuET separately induced SKOV-3 cell death at different dosages (Figs. 4A–4C). Interestingly, SKOV-3 cell death caused by CuET was more significant than that caused by DSF, and SKOV-3 cells were resistant to CuCl_2_ even at a high dose (Fig. 4D). Recently, a pan-cancer analysis revealed that ferredoxin 1 (Fdx1) is a key cuproptosis-related gene responsible for tumor immunity and drug sensitivity [31]. In the present study, the mRNA level of Fdx1 was found to be reduced after CuET treatment in SKOV-3 cells (Fig. 4E). Cuproptosis was also identified to destabilized the Fe-S cluster protein, causing proteotoxic stress and ultimately cell death [23]. Expression levels of genes of the Fe-S cluster, including LIAS, ACO-2, SDHB and POLD1 were also found to be significantly reduced after CuET treatment of SKOV-3 cells (Figs. 4F–4I). Moreover, the mRNA levels of LIAS, ACO-2, SDHB and POLD1 were not changed after DSF and CuCl_2_ treatment (Figs. 4F–4I). These results indicated that CuET induced ovarian tumor cell death though cuproptosis.

## Discussion

In oncology, drug repositioning has recently become a powerful alternative for developing novel anticancer drug [32,33]. Surprisingly, in the present study, results obtained using an *in vitro* small-molecule compound screening system, showed that an old alcohol-abuse drug DSF significantly reduced the expression of the anti-apoptosis marker B-cell lymphoma/leu kemia-2 (Bcl-2) while increasing the expression of the apoptosis markers Bcl2 associated X (Bax) and cleaved caspase-3; these results indicated that DSF promotes human epithelial ovarian cancer cells apoptosis. In addition, the DSF/Cu complex was found to inhibit human epithelial ovarian cancer cell viability in a time and dose-dependent manner. Apoptosis activation and promotion of tumor cell death are of great interest in overcoming tumor invasion. Indeed, this has led to the rapid discovery of new anticancer drugs capable of cell death stimulation, either individually or as a part of combinatorial therapies [10].

The brilliant idea of using copper to treat cancer was proposed decades ago. However, the use of copper for cancer treatment has not materialized in the clinic due to the limitation posed by the Cu transporter Ctr1 [34,35]. DSF has been reported to be a strong Cu chelator. Moreover, the DSF/copper complex is a significantly better inducer of ROS production in cancer cells compared with Cu [36,37]. In the present study, using the human epithelial ovarian cancer cell line SKOV-3, we investigated the cytotoxic effect of the DSF/Cu complex in human epithelial ovarian cancer both *in vitro* and *in vivo*. Coppper plays an essential role in cancer progression by acting as a co-factor for regulating the activities of many proteins and enzymes in cancer cells [38]. Therefore, Cu-based complexes have been identified as complementary agents for platinum-based cancer drugs with undesirable general toxicity. Understanding the fundamental role of several metal ions in cancer biological processes has garnered much attention. Several non-platinum metallodrugs such as zinc, gallilum, ruthenium and nickel-based compounds have also been studied for their anticancer potential [39–41]. Besides the DSF/Cu complex, DSF can also form the DSF/Na complex. However, DSF/Na showed litter effect on cell viability. Recently, it was suggested that nano-sized metalorganic frameworks (MOF) composed of metal ions and organic ligands are a great carrier material for drug delivery [42]. Therefore, in our future study, we will assess the ability of Cu-based MOF to enhance the antitumor effect of DSF.

Inflammatory cytokines play a crucial role in tumor growth and the tumor microenvironment. In the present study, we found that the level of proinflammatory cytokines including IL-1β, IL-6 and TNF-α were significantly increased in tumor-bearing mice after DSF treatment. Recent studies have suggested that tumor necrosis factor-α (TNF-α) promotes the expression of pro-inflammatory cytokines (IL-1β, IL-6 and TNF-α) in human epithelial ovarian tumors [43]. Moreover, ovarian tumor cells are known to secrete large amounts of TNF-α into the tumor microenvironment. Therefore, we speculated here, that the increased levels of the pro-inflammatory cytokines, IL-1β, IL-6 and TNF-α in tumor-bearing mice is dependent on the production of TNF-α by ovarian tumor cells. Besides IL-1β, IL-6 and TNF-α, recent studies have shown that prostaglandin E2, another pro-inflammatory cytokine, is over-expressed in various human epithelial malignancies [44]. Therefore, further studies are required for assessing the expression of other pro-inflammatory cytokines in a murine ovarian tumor model, and comparing the effects of DSF on the expression levels of these pro-inflammatory cytokines.

Besides assessing the expression levels of pro-inflammatory cytokines, the present study also explored the expression of anti-inflammatory cytokines in ovarian tumors. Both qPCR and ELISA results showed a reduced expression of anti-inflammatory cytokines including IL-4, IL-10 and TGF-β in murine ovarian tumor after DSF treatment. Although the target of DSF in the study was ovarian tumor cells, recent studies have also shown that DSF can interfere with frount-chemokine receptor via direct binding to a specific site of the chemokines receptor-binding domain of frount in macrophages [45]. Since macrophages are the main sources of anti-inflammatory cytokines, we speculate that DSF can also target tumor-associated macrophages in ovarian tumor, to promote the production of anti-inflammatory cytokines.

Further, the present study revealed that the ditocarbamate-copper complex CuET significantly promotes the apoptosis of human epithelial SKOV-3 cells. Over the past years, ovarian cancer treatment has mainly involved increasing DNA damage to activate tumor apoptosis pathway [46]. Olaparib was the first FDA-approved PARP inhibitor; it is used in BRCA 1/2-mutated ovarian cancer patients who have already received three or more chemotherapy regimens. Olaparib has achieved good clinical efficacy in treating ovarian cancer. However, only about 50% of ovarian cancers are homologous recombination deficient. Recent studies have shown that the combination of olaparib and proguanil increased apoptosis in ovarian cancer cells, more significantly than each agent alone. Therefore, in our future study, we will assess the effects of combination treatment with DSF and clinical drugs (e.g., olaparib or proguanil) on ovarian cancer.

Our study showed that DSF plus cupper significantly induced human epithelial SKOV-3 cell death. Various heavy metals can induce regulated cell death through different subroutines. A previous study published in Science found that intracellular copper accumulation triggers the aggregation of mitochondrial lipoylated proteins and the destabilization of Fe–S cluster proteins, leading to a unique type of cell death termed cuproptosis [23]. Recently, DSF exhibited a strong anti-immune response in systemic lupus erythematous [47,48]. Beyond its role in cell death, DSF was also an inhibitor of GSDMD and cell pyroptosis [49]. Activation of GSDMD has been identified in many non-infectious diseases [50–52]. So the role of GSDMD may be also an interesting target by DSF in ovarian cancer.

## Conclusion

The data generated in the present study highlight the role of CuET-mediated ROS production and cell cuproptosis in ovarian cancer. By using a drug reposition screening system, we found DSF significantly suppressed the viability of human epithelial SKOV-3 cells. Our results also highlight an essential role of CuET in Bax/Bcl-2/caspase-3 pathway and loss of Fe-S proteins. Given the significant unmet clinical needs of ovarian cancer patients, the therapeutic potential for the repurposing of this drug with copper as a means of treating ovarian cancer warrants furtherer study.

## Materials and Methods

### Materials

Dulbecco’s modified Eagle’s medium (DMEM), 1640 medium and diamidino-2-phenylindole (DAPI) were obtained from (Sigma Millipore, St. Louis, MO, USA). Fetal bovine serum (FBS) was acquired from GIBCO (Sigma Millipore, St. Louis, MO, USA). The BCA Protein Assay Kit was from Junyan Biotechnology (S0111A-96, Taiyuan, China). DSF (T0054) and Diethyldithiocarbamate-copper complex (CuET) was obtained from TOPSCIENCE (Shanghai, China). The Anti-Bax antibody was purchased from (2774, Cell signaling Technology, Danvers, MA, USA). The Anti-Bcl-2 antibody was purchased from (3498, Cell signaling Technology, Danvers, MA, USA). The Anti-cleaved caspase-3 antibody was purchased from (9661, Cell signaling Technology, Danvers, MA, USA). The anti-GAPDH antibody was obtained from (5174, Cell signaling Technology, Danvers, MA, USA).

### Cell culture

The human epithelial ovarian cancer cell lines SKOV3 (RRID:CVCL_0532), A2780 (RRID:CVCL_0134) and normal kidney cells HEK293T (RRID:CVCL_0063) were purchased from the Cell Bank of the Chinese Academy of Sciences (Shanghai, China). Cells were cultured in RPMI 1640 medium (SKOV3) or DMEM medium (A2780 and HEK293T) with 10% FBS and 1% penicillin/streptomycin. All cells were incubated at 37°C in a humidified 5% CO_2_.

### CCK8 assay for determining the effects of screened drugs on SKOV-3 cell viability

A total of 4 × 10^4^ SKOV-3 cells per well were seeded in the 96-well plates in 100 μL of 10% FBS medium. Cells were treated with compounds from anti-cancer compound libary (Topscience Co., Ltd., Shanghai, China) at 37°C. After 72 h, these cells were mixed with 10μl of CCK8 stock solution and incubated for 3 h at 37°C for assessing the effects of the screened drugs on SKOV-3 cell viability. The OD value of the cell suspensions were then measured at 450 nm. The data were obtained as follows: Viability (%) = [OD_450_ (Cells treated with indicated concentration of the drug in the presence of CCK8) − OD_450_ (Only medium)]/[OD_450_ (Cells treated with DMSO in the presence of CCK8) − OD_450_ (Only medium)] × 100.

### FACS analysis of SKOV-3 cell viability after treatment with screened drugs

A total of 4 × 10^4^ SKOV-3 cells per well were seeded in the 96-well plates in 100 μL of 10% FBS medium. Cells were treated with the screened small-molecule compounds having anti-cancer potential (Topscience Co., Ltd.) at 37°C. After 72 h, the cells were collected and suspended in binding buffer. They were then stained with 7-AAD for 15 min at 4°C in the dark at room temperature. The BD LSRFortessa X-20 instrument was used for data acquisition, and the FlowJo software was used for data analysis.

### FACS analysis of SKOV-3 cell apoptosis caused by screened drugs using Annexin V/PI staining

A total of 2 × 10^5^ SKOV-3 cells per well were seeded in the six-well plates in 2 mL of 10% FBS medium. Cells were seeded in six-well plates for 16–14 h and incubated with 5 μM CuET for 12 h. The cells were then collected and fixed in 500 μM Annexin V binding buffer containing 5 μL Annexin V-APC and 5 μL 7-AAD. After incubation at room temperature for 20 in the dark, 2 × 10^4^ cells were analyzed using fluorescence-activated cell sorter analysis. The experiments were repeated three times.

### Murine ovarian cancer xenograft model

Female NOD/SCID mice, 6–8 weeks old, were purchased from SLAC. All mice were maintained in a specific pathogen-free facility at the First Affiliated Hospital of Jinan University. Mice with matched age and sex were used for all experiments, and mice were randomly allocated to experimental groups. A total of 1.25 × 10^6^ SKOV-3 cells were subcutaneously injected into the NOD/SCID mice. After 2 weeks, the mice were divided into two groups: vehicle group and DSF group. In the DSF group, copper gluconate was administered to the mice each morning (8 am) and DSF was administered each evening (7 pm), mimicing a previously conducted clinical trial on the combined DSF and copper gluconate treatment of tumors involving the liver (NCT00742911) [16]. All animal experiments were approved by the Institutional Animal Care and Use Committee of First Affiliated Hospital of Jinan University. Tumor volume was estimated using the following formula: tumor volume = 0.5 × length × width^2^. The maximal tumor volumes measurements are in accordance with the Institutional Animal Care and Use Committee (IACUC) in Jinan University. Tumor and spleen were acquired from the mice. The sections were stained with H&E staining.

### Real-time quantitative PCR (q-PCR) analysis of cytokine mRNA expression

A total of 2 × 10^5^ SKOV-3 cells per well were seeded in the six-well plates in 2 mL of 10% FBS medium. A total of 2 × 10^5^ SKOV-3 cells per well were seeded in the six-well plates in 2 mL of 10% FBS medium. The method was performed as previously described. Total RNA was extracted using Trizol reagent following manufacturer’s instructions (9109, Takara, Japan), and reverse-transcribed into cDNA using PrimeScript^™^ RT Reagent Kit (RR036, Takara). qRT-PCR was performed with SYBR Green (B21703, Bimake, China). the relative quantity of mRNA transcripts of each gene was calculated by using the 2^−ΔΔct^ method [53], and β-actin served as the endogenous control for normalization. Sequences of the qPCR primers are as follows: *IL-1β*, Forward: 5′-GCAACTGTTCCTGAACTCAACT-3′, Reverse: 5′-ATCTTTTGGGGTCCGTCAACT-3′; *IL-6*, Forward: 5′-GAGTTGTGCAATGGCAATTCTG-3′, Reverse: 5′-GCAAGTGCATCATCGlTGTTCAT-3′; TNF-α, Forward: 5′-ATGGCTGCTCAAGGCTGGTC-3′, Reverse: 5′-AGGCTTTTCATGCTCAACACTAT-3′; *β-actin*, Forward: 5′-AGTGTGACGTTGACATCCGT-3′, Reverse: 5′-GCAGCTCAGTAACAGTCCGC-3′. *IL-4*, Forward: 5′-GGCATTTTGAACGAGGTCAC-3′, Reverse: 5′-AAATATGCGAAGCACCTTGG-3′; *IL-10*, Forward: 5′-TGAATTCCCTGGGTGAGAAG-3′, Reverse: 5′-TGGCCTTGTAGACACCTTGG-3′; *TGF-β*, Forward: 5′-GGAGGTTTATAAAATCGACATGC-3′, Reverse: 5′-GGCATATGTAGAGGTGCCATC-3′; *Fdx1*, Forward: 5′-CACCGGTCCACTTTATAAACCGTGA-3′, Reverse: 5′-AAACTCACGGTTTATAAAGTGGACC-3′; *LIAS*, Forward: 5′-CACCGCAGGAGTAAGACACTCCACA-3′, Reverse: 5′-AAACTGTGGAGTGTCTTACTCCTGC-3′; *SDHB*, Forward: 5′-ATCGATGGGACCCAGACAAG-3′, Reverse: 5′-ACATACATGTGTGGAAGAGGGT-3′; *POLD1*, Forward: 5′-GTCCCTCTCTGGCTACTTGC-3′, Reverse: 5′-ACAGAGAACCCCTCATCGGT-3′; *ACO2*, Forward: 5′-GTGGCGATGAGCCACTTTGA-3′, Reverse: 5′-CACAGTGGATGGTGGATGGC-3′; Data were normalized by the β-actin expression level in each sample. Thermal cycling was performed in a Bio-Rad CFX96 Touch (Bio-Rad Laboratories) under the following conditions: 95°C for 2 min followed by 40 cycles at 95°C for 5 s, and 60°C for 30 s. All samples were loaded in triplicates on 96-well plates.

### Enzyme linked immunosorbent assay (ELISA) for analyzing cytokine expression levels

Cells were collected after treatment as mentioned in the figure legend. ELISA was performed as per the protocols used in previous study. Briefly, cell supernatants were collected and ELISA was performed using as follows: 100 μl of cell supernatants were mixed with IL-1β, IL-6 and TNF-α standards (Biolegend) for 2 h at room temperature, washing was performed six times with wash buffer, incubation with 100 μl/well of purified anti-mouse antibodies was performed for 2 h at room temperature, washing was performed six times with wash buffer, incubation was performed with 100 μl/well of HRP for 1 h at RT, washing was performed six times with wash buffer, incubation was performed with 100 μl/well of 1 × TMB solution at RT, and the reaction was stopped with 100 μl/well of stop solution. Finally, data were collected at 450 nM and normalized to those for the vehicle control (Synergy HTX).

### Western blot

SKOV-3 cells treated with DSF (5 μM) were seeded into 6-well plates, harvested after 12 h, and lysed using RIPA lysis buffer supplemented with a protease and phosphatase inhibitor cocktail (Topscience Co., Ltd.). The obtained total protein was loaded onto a 10% polyacrylamide gel for separation using SDS-PAGE, and separated proteins were then transferred onto a PVDF membrane at 250 mA for 80 min. The membrane was blocked at room temperature with 5% fat-free milk for 1 h and incubated at 4°C overnight with the following primary antibodies.

Bax (dilution: 1:1000), Bcl-2 (dilution: 1:1000), Cleaved caspase-3 (dilution: 1:1000), GAPDH (dilution: 1:3000) from Cell Signaling Technology. The membrane was washed three times in the TBST and probed with secondary antibodies (Proteintech, China). Finally, the membrane was imaged by imaging system (Tanon, Shanghai, China). In each membrane, the gray value of proteins was calculated by ImageJ (NIH, V2.0.0), and relative to the gray value of GAPDH.

### Cell cycle analysis using flow cytometry

A total of 4 × 10^4^ SKOV-3 cells per well were seeded in the 96-well plates in 100 μL of 10% FBS medium. After DSF treatment, SKOV-3 cells were fixed in 75% ethanol for 12 h. The cells were then collected and suspended in binding buffer, following which they were stained with 7-AAD for 15 min at 4°C in the dark at room temperature. Then, the cells were stained with propidium iodide (Beyotime) for cell cycle analysis. Finally, the percentage of cells in each cell cycle phase (G0/G1, S and G2/M) was determined. Data were acquired with BD FACSDiva (v8.0.2) on a BD Fortessa X20 (BD Biosciences) and analyzed using FlowJo software (Becton, Dickinson & Company, CA, USA)

### Measurement of intracellular ROS levels

The intracellular ROS levels were measured as previously described [54]. Briefly, ROS levels were measured using a Reactive Oxygen Species Assay Kit (Beyotime Biotechnology, China) based on 2′, 7′-dichlorofluorescein-diacetate (DCFH-DA). This kit detects ROS based on the principle that DCFH is oxidized by intracellular ROS to the fluorescent compound dichlorofluorescein (DCF). Cells were seeded in 96-well plates at a density of 2 × 10^4^ and exposed to different concentrations of DSF for different time durations. Following treatment, the cells were incubated with DCFH-DA for 20 min at 37°C and then observed using confocal microscopy (Leica sp8); fluorescence was measured at a 488 nm excitation wavelength and 555 nm emission wavelength using a fluorescence spectrophotometer.

### Statistical analysis

All data were statistically analyzed using the SPSS version 20.0 (SPSS Inc., Chicago, IL, USA) and Graphpad Prism v8.0 software. Data are expressed as mean ± SD (standard deviation). Unpaired two-tailed Student’s *t* test was used to analyze the differences between two groups. Comparisons among multiple groups were analyzed with one-way analysis of variance followed by the Tukey test. Survival curves were estimated for each group, considered separately, using the Kaplan–Meier method and compared statistically using the log rank test. *p* < 0.05 was considered statistically significant.

## Data Availability

The raw date supporting the conclusion of this article will be made available by the authors, without undue reservation.
